# The value of the atherogenic index of plasma in non-obese people with non-alcoholic fatty liver disease: a secondary analysis based on a cross-sectional study

**DOI:** 10.1186/s12944-020-01319-2

**Published:** 2020-06-23

**Authors:** Bu-yuan Dong, Yu-qing Mao, Zheng-yang Li, Fu-jun Yu

**Affiliations:** 1 Department of Gastroenterology, the First Affiliated Hospital of Wenzhou Medical University, Nanbaixiang, Ouhai District, Wenzhou, Zhejiang PR China; 2 Department of Gastroenterology, Shanghai First People’s Hospital, School of Medicine, Shanghai Jiao Tong University, Shanghai, China; 3 Department of Gastroenterology, Jinshan Hospital of Fudan University, Jinshan, Shanghai, 201508 China

**Keywords:** Non-alcoholic fatty liver disease, Atherogenic index of plasma, Receiver operating characteristic curve, Non-obese patients, Odds ratio, Risk factor, Correlation

## Abstract

**Background and objectives:**

The atherogenic index of plasma (AIP) is elevated in fatty liver disease, but its value in non-obese people with non-alcoholic fatty liver disease (NAFLD) is unclear. This study aimed to investigate the relationship between AIP and NAFLD as well as to determine whether AIP might be used as an indicator of NAFLD in non-obese individuals.

**Methods:**

The present study involved non-obese Chinese and Japanese participants. Risk factors are evaluated using univariate and multivariate analysis. The performance of risk factors was compared according to the area under the receiver operating characteristic curve.

**Results:**

In the unadjusted model, the odds ratio (OR) for every 1 standard deviation (SD) increase in AIP was 52.30. In adjusted models I and II, the OR for every 1 SD increase in AIP was 36.57 and 50.84, respectively. The area under the receiver operating characteristic curve for AIP was 0.803 and 0.802 in the development and validation groups, respectively. The best cut-off value of AIP for discrimination between NAFLD and non-NAFLD was 0.005 in the Chinese group and − 0.220 in the Japanese group.

**Conclusions:**

AIP and NAFLD are positively correlated in Chinese and Japanese populations. Therefore, AIP can be used as a new screening indicator for non-obese people with NAFLD in different nations.

## Introduction

Non-alcoholic fatty liver disease (NAFLD) is a common chronic liver disease worldwide. The disease may progress to liver cirrhosis and liver cancer [1, 2]. Obesity is an important risk factor for NAFLD [3]. Liver histology and non-invasive fibrosis tests suggest that some non-obese people may also have non-alcoholic steatohepatitis and advanced fibrosis despite having a body mass index (BMI) within the normal range [4–6].

The plasma atherosclerosis index (AIP) is a quantitative indicator used to evaluate blood lipid levels. AIP has good predictive value for dyslipidemia diseases such as diabetics, atherosclerosis and heart disease [7–9]. AIP has important predictive value not only for cardiovascular disease but also for hyperuricemia [10]. Several studies have reported a positive correlation between high levels of AIP and obesity [11], and have shown that AIP has better discriminatory ability for NAFLD in obese people [12]. However, there has been little relevant research on non-obese people. More attention should be paid to non-obese people with NAFLD, who often think that a fatty liver is unlikely in the absence of obesity.

This study aimed to investigate the relationship between AIP and NAFLD in non-obese people and to demonstrate that AIP is an independent risk factor for NAFLD in non-obese individuals.

## Materials and methods

### Data sources

Data in the study came from the Dryad Digital Repository website (www.datadryad.org), which allows users to download raw data free of charge. These data are anonymous. According to the Dryad Terms of Service, researchers may apply these data in secondary analysis without infringing on the authors’ rights. In the study, the Chinese data came from the following source [13]: Association of low-density lipoprotein cholesterol within the normal range and NAFLD in the non-obese Chinese population: a cross-sectional and longitudinal study. Dataset website: 10.5061/dryad.1n6c4. The Japanese data came from the following source [14]: Ectopic fat obesity presents the greatest risk for incident type 2 diabetes: a population-based longitudinal study. Dataset website: 10.5061/dryad.8q0p192. Variables included in the Chinese database file were as follows: age, sex, γ-glutamyltranspeptidase (GGT), alanine aminotransferase (ALT), aspartate aminotransferase (AST), total protein (TP), albumin (ALB), globulin (GLB), total bilirubin (TB), direct bilirubin (DBIL), blood urea nitrogen (BUN), creatinine (Cr), estimated glomerular filtration rate (eGFR), uric acid (UA), low-density lipoprotein cholesterol (LDL-c), fasting plasma glucose (FPG), high-density lipoprotein cholesterol (HDL-c), total cholesterol (TC), triglyceride (TG), BMI, AIP and fatty liver. Variables included in the Japanese database file were as follows: age, GGT, ALT, AST, HDL-c, BMI, AIP and sex.

### Study design and participants

For the Chinese study population, participants took part in health examination at the First Affiliated Hospital of Wenzhou Medical University between January 2010 to December 2014. A total of 78,304 participants were recruited and selected on the basis of the following exclusion criteria: (1) a lack of required data; (2) excess alcohol consumption (more than 20 g per day for men or 10 g per day for women); (3) known liver disease; (4) BMI ≥ 25 kg/m^2^; (5) LDL-c > 3.12 mmol/L; and (6) use of antihypertensive agents, antidiabetic agents or lipid-lowing agents. Diagnosis of fatty liver was performed in accordance with the ultrasound diagnostic criteria of the Chinese Liver Disease Association [15]. AIP was the base-10 logarithm of the ratio of the concentration of TG to HDL-c in concentration units of mmol/L, according to the formula AIP = log (TG/HDL-c) [16]. BMI was calculated as the weight in kilograms divided by the height in m^2^, and represented an index of body fat. All biochemical values were analyzed with an automatic measurement analyzer (Abbott) according to standard methods. Among the research population in Japan, a total of 12,932 participants who had undergone medical examination at Murakami Memorial Hospital between 2004 and 2015 were recruited and selected according to the following exclusion criteria: (1) a lack of important data; (2) known liver disease; (3) alcohol intake exceeding 60 g per day for men or 40 g per day for women; (4) drug use; (5) fasting blood glucose ≥6.1 mmol/L; and (6) BMI ≥ 25 kg/m^2^. Because this study was a secondary study, and the data were anonymous, no informed consent was required. Specific details are given in the original report [14].

### Diagnosis of NAFLD by ultrasonography

NAFLD is defined by the diffusion enhancement of near-field echo and the gradual attenuation of far-field echo in the liver area (stronger than that in the kidney and spleen area). One of the following conditions must be present: (1) reduced blood flow signal but normal blood flow distribution; (2) mild to moderate hepatomegaly, with round and blunt borders;(3) unclear or incomplete envelope of the right liver lobe and the diaphragm muscle;(4) unclear liver cavity structure. NAFLD is diagnosed with abdominal ultrasonography performed by trained technicians.

### Statistical analysis

The overall statistical analysis in this study consisted of five steps. First, in the Chinese study, the population was divided into a development group and validation group in a 7:3 ratio. Continuous variables are evaluated by calculating the means ± standard deviations (SD) (normal distribution) or medians (quartiles) (skewed distribution), and categorical variables are evaluated by calculating frequencies or percentages. Differences across groups were analyzed using One-way ANOVA (normal distribution), Kruskal-Wallis H (skewed distribution) test and chi-square test (categorical variable). Second, risk factors in the development group were analyzed using univariate and multivariate regression analysis. Independent variables were tested for collinearity and were excluded with the variance inflation factor (VIF) ≥ 10. Collinear VIF = 1/(1-R^2^) [17]. The subgroups were grouped with a linear regression model. Third, according to the recommendation of the STROBE statement [18], the results of the unadjusted, minimally adjusted analysis and fully adjusted analysis are reported. Fourth, the area under the receiver operating characteristic (AUROC) curve of each predictor was used to compare the predictive utility. Fifth, box plots were used to intuitively reflect the predictive value of cut-off. All tests were two-sided. A *P*-value < 0.05 was considered statistically significant. The R (version 3.4.3, The R Foundation; http://www.r-project.org) statistical package and GraphPad Prism (version 8.0; GraphPad Software) were used for statistical analysis.

## Results

### Participants’ baseline data

As shown in Table 1, 78,304 Chinese participants were included in the study. The development group comprised 23,265 women and 31,608 men with an average age of 44.6 years. The average age, TP, ALB, GLB, TB, BUN, Cr, eGFR, UA, FPG, TC, LDL-c and BMI were greater in patients with than without NAFLD. The median GGT, ALT, AST, TG and AIP were greater in patients with than without NAFLD. The same trend was observed in the validation group. Among the Japanese participants, 11,598 people with NAFLD and 1334 people without NAFLD were included. The age, GGT, ALT, BMI, AIP, TG and FPG were greater in patients with than without NAFLD (Table S1).

**Table 1:** Baseline Characteristics of the Chinese Study Participants

Characteristic	Development Group			Validation Group		
	Non-NAFLD	NAFLD	*P*-value	Non-NAFLD	NAFLD	*P*-value
No. of participants	45,969	8904		19,742	3689	
Age	43.7 ± 15.6	49.4 ± 13.5	< 0.001	43.6 ± 15.6	49.7 ± 13.8	< 0.001
GGT (U/L)	19.0 (15.0,27.0)	33.0 (24.0,51.0)	< 0.001	19.0 (15.0,27.0)	33.0 (24.0,51.0)	< 0.001
ALT (U/L)	15.0 (12.0,21.0)	24.0 (18.0,34.0)	< 0.001	15.0 (12.0,21.0)	24.0 (17.0,34.0)	< 0.001
AST (U/L)	20.0 (18.0,24.0)	24.0 (20.0,28.0)	< 0.001	20.0 (18.0,24.0)	23.0 (20.0,28.0)	< 0.001
TP (U/L)	73.6 ± 4.4	74.3 ± 4.3	< 0.001	73.5 ± 4.4	74.3 ± 4.4	< 0.001
ALB (U/L)	44.5 ± 2.8	45.0 ± 2.7	< 0.001	44.5 ± 2.8	45.0 ± 2.7	< 0.001
GLB (U/L)	29.0 ± 4.0	29.3 ± 4.1	< 0.001	29.0 ± 3.9	29.4 ± 4.1	< 0.001
TB (mmol/L)	12.5 ± 5.0	12.6 ± 4.9	0.012	12.5 ± 5.2	12.6 ± 4.9	0.004
DBIL (mmol/L)	1.8 (1.3,2.4)	1.8 (1.4,2.5)	< 0.001	1.8 (1.3,2.4)	1.9 (1.4,2.5)	< 0.001
BUN (mmol/L)	4.4 ± 1.4	4.7 ± 1.3	< 0.001	4.4 ± 1.4	4.7 ± 1.4	< 0.001
Cr (μmol/L)	81.2 ± 24.2	86.9 ± 18.5	< 0.001	81.3 ± 25.5	88.1 ± 23.9	< 0.001
eGFR (mL/min/1.73 m2)	88.2 ± 23.1	88.0 ± 22.9	< 0.001	88.1 ± 23.0	87.9 ± 23.1	< 0.001
UA (μmol/L)	278.5 ± 85.7	345.7 ± 86.8	< 0.001	279.1 ± 85.6	347.6 ± 89.0	< 0.001
FPG (mmol/L)	5.2 ± 0.8	5.7 ± 1.4	< 0.001	5.2 ± 0.8	5.7 ± 1.3	< 0.001
TC (mmol/L)	4.5 ± 0.7	4.8 ± 0.8	< 0.001	4.5 ± 0.7	4.8 ± 0.8	< 0.001
TG (mmol/L)	1.0 (0.8,1.4)	1.8 (1.3,2.6)	< 0.001	1.0 (0.8,1.4)	1.8 (1.4,2.6)	< 0.001
HDL-c (mmol/L)	1.5 ± 0.3	1.2 ± 0.3	< 0.001	1.5 ± 0.3	1.2 ± 0.3	< 0.001
LDL-c (mmol/L)	2.3 ± 0.5	2.4 ± 0.5	< 0.001	2.3 ± 0.5	2.4 ± 0.5	< 0.001
BMI (kg/m^2^)	21.3 ± 2.1	23.4 ± 1.2	< 0.001	21.3 ± 2.0	23.4 ± 1.2	< 0.001
AIP	−0.1 (− 0.3,0.0)	0.2 (0.0,0.4)	< 0.001	− 0.1 (− 0.3,0.0)	0.2 (0.0,0.4)	< 0.001
Sex			< 0.001			< 0.001
Female	21,650 (47.1%)	1615 (18.1%)		9289 (47.1%)	654 (17.7%)	
Male	24,319 (52.9%)	7289 (81.9%)		10,453 (52.9%)	3035 (82.3%)	

### Univariate and multivariate analysis in the development group

The univariate and multivariate analysis results are shown in Table 2. Univariate logistic regression results indicated that men were at higher risk of NAFLD than women. Higher age, GGT, ALT, AST, TP, ALB, GLB, TB, DBIL, BUN, Cr, UA, FPG, TC, TG, LDL-c, BMI and AIP were found in patients with than without NAFLD, thus indicating that these variables are risk factors in the progress of fatty liver disease. eGFR and HDL-c were lower in patients with than without NAFLD, thus indicating that these variables are protective factors. To exclude the mutual influence of these variables, variables with VIF < 10 were analyzed with a multivariate regression model. Age, GGT, ALT, ALB, DBIL, UA, FPG, LDL-c, BMI and AIP were independent factors positively correlated with the progress of fatty liver disease, among which AIP was the strongest factor.

**Table 2:** Results of univariate and multivariate regression analysis in development group

Exposure	Univariate			Multivariate		
	OR	(95%CI)	*P*-value	OR	(95%CI)	*P*-value
Sex						
Female	Reference	Reference		Reference		
Male	4.02	(3.80, 4.25)	< 0.001	0.77	(0.71, 0.84)	< 0.001
Age	1.02	(1.02, 1.02)	< 0.001	1.01	(1.01, 1.02)	< 0.001
GGT (U/L)	1.02	(1.02, 1.02)	< 0.001	1.00	(1.00, 1.01)	< 0.001
ALT (U/L)	1.04	(1.04, 1.04)	< 0.001	1.03	(1.03, 1.03)	< 0.001
AST (U/L)	1.03	(1.03, 1.04)	< 0.001	0.97	(0.96, 0.97)	< 0.001
TP (U/L)	1.04	(1.03, 1.05)	< 0.001			
ALB (U/L)	1.07	(1.06, 1.08)	< 0.001	1.06	(1.05, 1.07)	< 0.001
GLB (U/L)	1.01	(1.01, 1.02)	< 0.001	1.00	(0.99, 1.01)	
TB (mmol/L)	1.01	(1.00, 1.01)	< 0.05	0.99	(0.98, 0.99)	< 0.001
DBIL (mmol/L)	1.07	(1.04, 1.09)	< 0.001	1.10	(1.07, 1.14)	< 0.001
BUN (mmol/L)	1.13	(1.11, 1.15)	< 0.001	0.94	(0.92, 0.96)	< 0.001
Cr (μmol/L)	1.01	(1.01, 1.01)	< 0.001			
eGFR (mL/min/1.73 m2)	0.99	(0.98, 0.99)	< 0.001			
UA (μmol/L)	1.01	(1.01, 1.01)	< 0.001	1.00	(1.00, 1.00)	< 0.001
FPG (mmol/L)	1.55	(1.51, 1.59)	< 0.001	1.23	(1.20, 1.26)	< 0.001
TC (mmol/L)	1.70	(1.64, 1.75)	< 0.001	1.06	(0.99, 1.14)	
TG (mmol/L)	2.62	(2.55, 2.69)	< 0.001	0.92	(0.88, 0.96)	< 0.001
HDL-c (mmol/L)	0.11	(0.10, 0.12)	< 0.001			
LDL-c (mmol/L)	2.18	(2.07, 2.29)	< 0.001	1.29	(1.16, 1.42)	< 0.001
BMI (kg/m^2^)	2.06	(2.02, 2.09)	< 0.001	1.69	(1.65, 1.72)	< 0.001
AIP	52.30	(47.64, 57.41)	< 0.001	15.65	(12.85, 19.05)	< 0.001

### Independent effect of AIP on the incidence of NAFLD

**Fig. 1:**
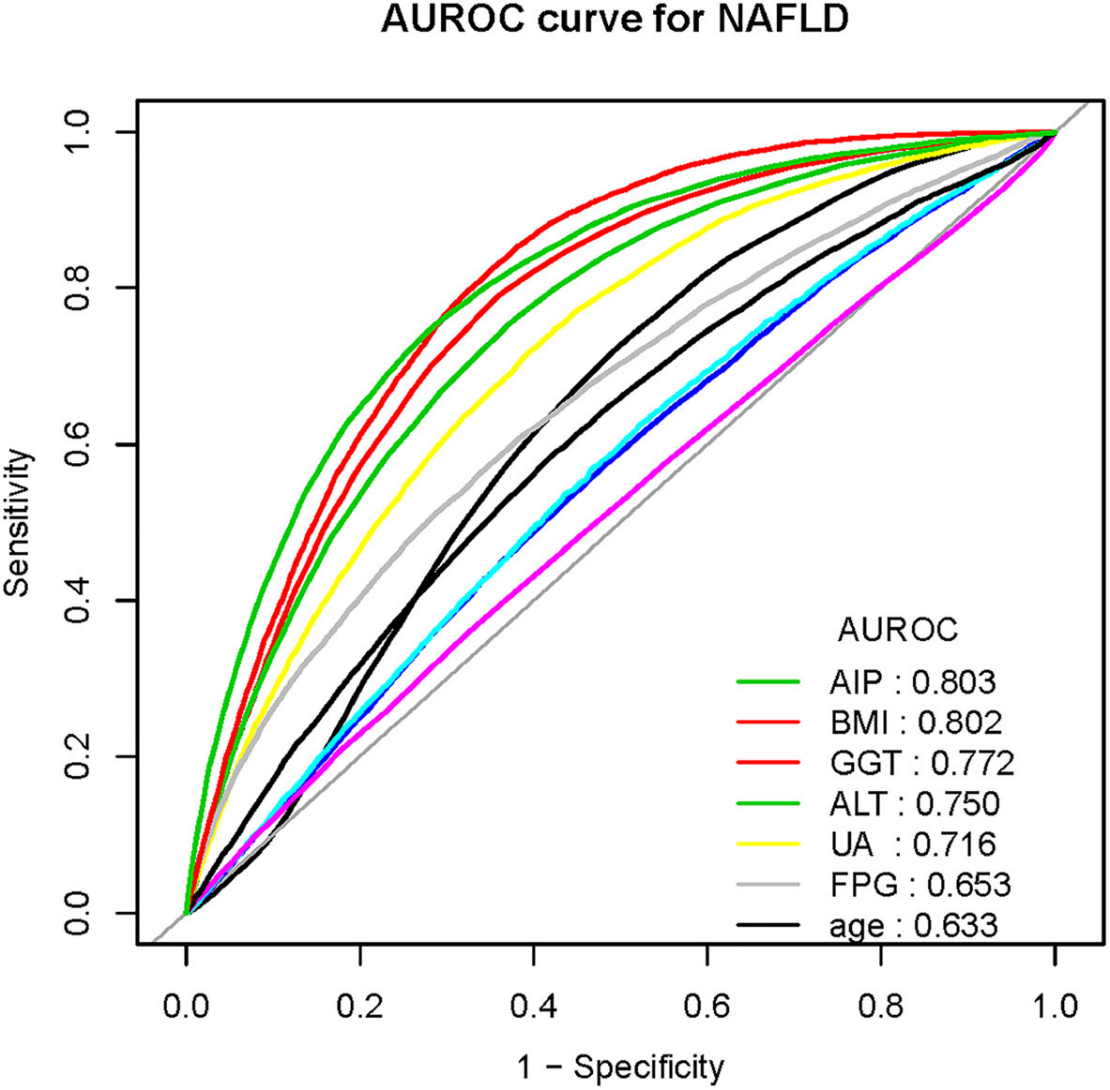
AUROC curve of all risk factors in the development group

**Fig. 2:**
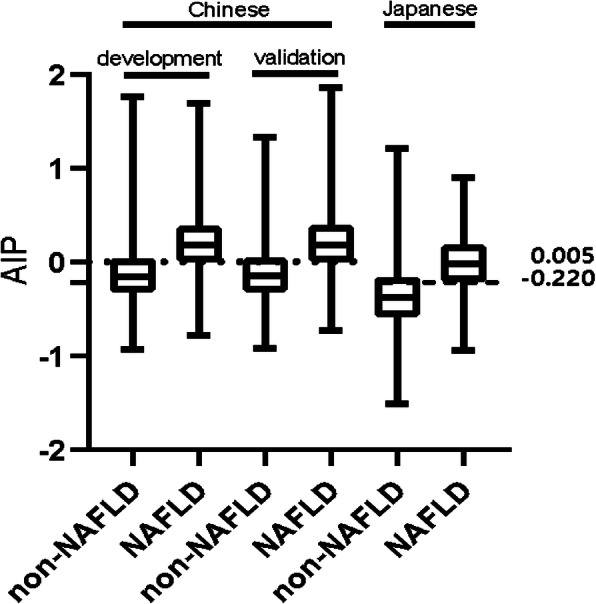
AIP for participants with or without NAFLD in different populations. The top and bottom edges of each box represent the third and first quartiles, respectively. Black bar within the boxes represents median value. Two horizontal lines represent AIP cut-off values of 0.005 and − 0.220

As shown in Table 3, the unadjusted odds ratio (OR) for every 1 SD increase in AIP was 52.30 (OR:52.30, 95% CI, 47.64–57.41, *P* <  0.001). After adjustment for age, GGT, ALT, ALB, DBIL, UA, FPG, LDL-C and BMI (adjusted model I), the OR for every 1 SD increase in AIP was 36.57 (OR: 36.57, 95% CI: 33.20–40.29; *P* <  0.001). After full adjustment for sex, age, GGT, ALT, AST, TP, ALB, GLB, TB, DBIL, BUN, Cr, UA, FPG, TC, TG, LDL-c and BMI (adjusted model II), the OR for every 1 SD increase in AIP was 50.84 (OR: 50.84, 95% CI: 38.22–67.63; *P* <  0.001). With OR > 1 in three models, AIP was positively correlated with NAFLD, and the results were stable (Fig.S1). For further sensitivity analysis, AIP was converted to a categorical variable, and the results obtained were consistent (Table 3). Similar results were seen in the Japanese population (Table S2). Because AIP, age, GGT, ALT, ALB, eGFR, DBIL, UA, FPG, LDL-c and BMI were independent risk factors for NAFLD, their diagnostic performance for NAFLD were evaluated. AIP, which had the highest AUROC among these indicators, had the best discrimination capacity (AUROC: 0.803, 95% CI: 0.798–0.808) in the development group (Fig.1 and Table 4), whereas DBIL had the worst performance (AUROC: 0.516, 95% CI: 0.509–0.523). In the validation group, BMI performed the best (AUROC: 0.808, 95% CI: 0.801–0.814), and AIP ranked second (AUROC: 0.802, 95% CI: 0.795–0.810). AIP had also the best discrimination ability (AUROC: 0.798, 95% CI: 0.787–0.810) in the Japanese group (Fig.S2 and Table S3). Then the best cut-off value according to the maximum Youden index of the AUROC was determined. As shown in Fig.2, the best cut-off value of AIP in discriminating between NAFLD and non-NAFLD was 0.005 in the Chinese group and was − 0.220 in the Japanese group.

**Table 3:** Effect modification of WHTR on incidence of NAFLD

Variable	Unadjusted		Adjusted Model I		Adjusted Model II	
	OR (95%CI)	*P*-value	OR (95%CI)	*P*-value	OR (95%CI)	*P*-value
WHTR (Per SD)	52.30(47.64–57.41)	< 0.001	36.57 (33.20–40.29)	< 0.001	50.84 (38.22–67.63)	< 0.001
WHTR (quartile)						
Q1	Reference		Reference		Reference	
Q2	3.18 (2.78–3.63)	< 0.001	2.67 (2.34–3.05)	< 0.001	1.80 (1.56–2.08)	< 0.001
Q3	7.91 (6.98–8.96)	< 0.001	5.88 (5.18–6.68)	< 0.001	2.74 (2.37–3.18)	< 0.001
Q4	28.67 (25.41–32.35)	< 0.001	19.64 (17.36–22.23)	< 0.001	5.04 (4.26–5.95)	< 0.001
*P* for trend	< 0.001		< 0.001		< 0.001	

Model I adjusted for sex, age

Model II adjusted for sex, age, GGT, ALT, AST, TP, ALB, GLB, TB, DBIL, BUN, Cr, eGFR, UA, FPG, TC, TG, HDL-c, LDL-c, BMI

*CI* Confidence interval.SD = 0.29

**Table 4:** ROC analysis for different continuous predictors in development and validation groups

	Development group					Validation group				
	AUROC	95%CI	Cut-off pot	Specificity	Sensitivity	AUROC	95%CI	Cut-off pot	Specificity	Sensitivity
Age	0.633	0.627–0.638	40.500	0.511	0.717	0.637	0.629–0.646	40.500	0.512	0.724
ALT	0.750	0.745–0.756	17.500	0.623	0.759	0.746	0.738–0.754	17.500	0.626	0.743
GGT	0.772	0.768–0.777	22.500	0.639	0.792	0.772	0.765–0.780	23.500	0.672	0.757
FPG	0.653	0.646–0.659	5.195	0.615	0.610	0.651	0.641–0.661	5.225	0.640	0.578
ALB	0.558	0.551–0.564	44.650	0.517	0.573	0.556	0.546–0.566	44.650	0.523	0.561
eGFR	0.562	0.556–0.569	67.165	0.479	0.625	0.577	0.567–0.587	64.485	0.569	0.554
DBIL	0.516	0.509–0.523	2.150	0.676	0.359	0.530	0.519–0.540	1.850	0.547	0.508
UA	0.716	0.710–0.721	295.500	0.609	0.716	0.717	0.708–0.725	291.500	0.589	0.736
LDL-c,	0.605	0.599–0.612	2.375	0.5740	0.591	0.604	0.594–0.614	2.385	0.584	0.579
BMI	0.802	0.798–0.807	22.335	0.647	0.826	0.808	0.801–0.814	22.285	0.645	0.836
AIP	0.803	0.798–0.808	0.005	0.715	0.752	0.802	0.795–0.810	0.025	0.735	0.733

The unit is mmol/L: HDL-c, LDL-c, GGT, DBIL, FPG and UA; The unit is U/L: ALT and ALB

### Subgroup analysis

The subgroup analysis results are shown in Table 5. The interactions were found to be significant for sex, age, AST, TB, BUN, UA, TC, TG, HDL-c, GGT, ALT, eGFR, FPG and BMI (*P* <  0.01), whereas the tests for interactions were not statistically significant for ALB, GLB, DBIL and LDL-c (*P* > 0.05). Although all variables were risk factors, they did not affect the correlation between AIP and NAFLD. Compared with that of patients over 60 years old, the AIP in patients under 60 years old was associated with higher risks of NAFLD (OR: 61.03 VS. 25.67). Similar results were found in female and male patients (OR: 92.43 VS. 29.11).

**Table 5:** Subgroup analysis of the association between AIP and NAFLD

	N	OR	95%CI	*P* value	*P* for interaction
Sex					< 0.001
Female	23,265	92.43	75.30–113.45	< 0.001	
Male	31,608	29.11	26.10–32.46	< 0.001	
Age (years)					< 0.001
< 60	45,394	61.03	54.95–67.78	< 0.001	
> = 60	9479	25.67	20.87–31.56	< 0.001	
AST (U/L)					< 0.001
< 21.500	30,411	56.93	49.12–65.97	< 0.001	
> = 21.500	24,462	37.13	32.88–41.94	< 0.001	
TB (mmol/L)					< 0.001
< 9.750	17,138	38.38	32.79–44.91	< 0.001	
> = 9.750	37,735	63.29	56.35–71.08	< 0.001	
BUN (mmol/L)					< 0.001
< 4.095	23,047	78.67	66.96–92.43	< 0.001	
> = 4.095	31,826	40.22	35.87–45.09	< 0.001	
UA (mmol/L)					< 0.001
< 295.500	30,509	68.38	57.90–80.76	< 0.001	
> = 295.500	24,364	25.37	22.55–28.55	< 0.001	
TC (mmol/L)					0.001
< 4.525	27,855	59.30	50.97–69.00	< 0.001	
> = 4.525	27,018	41.63	36.97–46.88	< 0.001	
TG (mmol/L)					< 0.001
< 1.395	36,755	98.11	75.21–127.96	< 0.001	
> = 1.395	18,118	14.98	12.80–17.53	< 0.001	
Total	54,873	25.33	22.09–29.04	< 0.001	
HDL-c (mmol/L)					< 0.001
< 1.335	24,257	37.46	32.79–42.80	< 0.001	
> = 1.335	30,616	131.01	107.80–159.22	< 0.001	
GGT (U/L)					< 0.001
< 22.5	31,203	48.1	40.0–57.8	< 0.001	
> = 22.5	23,670	29.1	18.6–23.4	< 0.001	
ALT (U/L)					< 0.001
< 17.5	30,801	47.1	39.9–55.6	< 0.001	
> = 17.5	24,072	28.7	25.5–32.2	< 0.001	
ALB (U/L)					0.656
< 44.65	27,585	49.8	43.4–57.2	< 0.001	
> = 44.65	27,288	52.0	45.8–59.0	< 0.001	
GLB (U/L)					0.304
< 29.15	28,786	54.8	48.1–62.5	< 0.001	
> = 29.15	26,087	49.7	43.5–56.8	< 0.001	
DBIL (mmol/L)					0.490
< 2.15	36,777	55.4	49.4–62.1	< 0.001	
> = 2.15	18,096	51.6	43.9–60.7	< 0.001	
eGFR					< 0.01
< 67.165	29,522	44.8	39.6–50.6	< 0.001	
> = 67.165	25,351	60.0	51.9–69.3	< 0.001	
FPG (mmol/L)					< 0.001
< 5.195	31,735	61.8	53.6–71.2	< 0.001	
> = 5.195	23,138	35.8	31.6–40.6	< 0.001	
LDL-c (mmol/L)					0.678
< 2.375	30,025	51.3	45.1–58.3	< 0.001	
> = 2.375	24,848	49.3	42.9–56.6	< 0.001	
BMI (kg/m2)					< 0.001
< 24	46,508	51.2	45.8–57.3	< 0.001	
> = 24	8365	17.0	14.2–20.4	< 0.001	

## Discussion

The purpose of this study was to analyze the relationship between AIP and NAFLD as well as to verify the diagnostic value of AIP in non-obese patients with NAFLD. AIP has been found to be an independent risk factor for NAFLD in non-obese patients through univariate and multivariate regression analysis, in agreement with results in obese patients [12]. Here, it was found that AIP was positively correlated with NAFLD in non-obese Chinese and Japanese patients. Subgroup analysis confirmed that many variables did not affect the positive correlation between AIP and NAFLD. Finally, AIP had better diagnostic value than the other variables in Chinese and Japanese patients, thus suggesting that AIP is applicable to different regions and ethnicities as a diagnostic indicator. However, the best cut-off value of AIP in discriminating between NAFLD and non-NAFLD differed between Chinese and Japanese patients, thus demonstrating that AIP has different standards for different regions and ethnicities. This research provides a reference for future health examinations.

Previous studies have focused mainly on obese patients with BMI above 25, thus often causing people with normal BMI to ignore their eating habits [19]. Dietary habits may play a major role in the development of NAFLD in non-obese people. For example, excessive intake of saturated fat, fructose, sucrose and refined carbohydrates and low intake of n-3 polyunsaturated fatty acids, natural antioxidants (fruits and vegetables) and dietary fiber can promote the accumulation of triglycerides in the liver, thus increasing the prevalence of NFALD [20]. Therefore, These results may be alarming for people with BMI in a normal range. Because some studies have indicated that the prevalence of NAFLD in non-obese individuals varies greatly across regions [21, 22], this study included Chinese and Japanese patient populations to exclude regional disparities. The positive correlation between AIP and NAFLD may reflect the following findings: 1. Dysfunctional, expanded and inflamed adipose tissue may be associated with NAFLD in people with normal-range BMI [23]. AIP is part of traditional lipid profiles, but it is better than traditional pro-atherogenic lipid profiles [24]. AIP as an excellent combination factor may have good performance in the diagnosis of NAFLD. 2. Insulin resistance is an independent risk factor for NAFLD in non-obese people [25]. AIP is an independent predictor of insulin resistance [26–29]. Therefore, on the basis of previous studies and current findings, it is concluded that AIP is correlated with NAFLD. 3. NAFLD is characterized by the accumulation of triacylglycerol in the liver, in addition to increased oxidative stress and inflammation [30].

Previous research has indicated that TG/HDL-c can be used as predictor of NAFLD in non-obese people [31]. This study confirmed the previous findings and provides a new reference for clinical medicine. Through subgroup analysis, it was found that AIP and NAFLD are more closely associated in females than males, in agreement with previous results [31]. Unexpectedly, AIP was more closely associated with NAFLD in non-obese people under than over 60 years of age, a finding that has not been reported in the previous literature. This result may be associated with slower metabolism in older people. However, the specific mechanism must be explored in the future.

### Study strengths and limitations

The study has several strengths. First, this research was a large sample, multi-region and multi-ethnic study. Second, this was a retrospective study with many confounding factors, in which strict statistics was used to minimize residual confounding. Third, the authors identified effective cut-off points for AIP in Chinese and Japanese populations, thus providing data support for clinical diagnosis.

There were, however, several limitations to this study. First, because it was a secondary study, information on the lifestyles and eating habits of the participating population was not collected. Second, this study focused on the relationship between AIP and NAFLD, without further evaluation of other variables.

## Conclusion

The results indicate that AIP and NAFLD are positively correlated in Chinese and Japanese populations. Therefore, AIP may be used as a new screening indicator for non-obese people with NAFLD in different nations, thus providing a reference for clinical work.

## Supplementary information

**Additional file 1: Table S1.** Baseline Characteristics of the Japanese Study Participants. **Table S2.** Effect of AIP on the incidence of NAFLD in Japanese. **Table S3.** AUROC analysis for different continuous predictors in Japanese groups. **Figure S1.** The relationship between AIP and NAFLD. **Figure S2.** AUROC curve of all risk factors in the Japanese group.

## Data Availability

Data can be downloaded from the ‘DATADRYAD’ database (www.datadryad.org).

## Abbreviations

GGT: γ-glutamyltranspeptidase
ALT: Alanine aminotransferase
AST: Aspartate aminotransferase
TP: Total protein
ALB: Albumin
GLB: Globulin
BUN: Blood urea nitrogen
TB: Total bilirubin
DBIL: Direct bilirubin
Cr: Creatinine
eGFR: Estimated Glomerular Filtration Rate
LDL-c: Low-density lipoprotein cholesterol (LDL-c)
UA: Uric acid
TG: Triglyceride
FPG: Fasting plasma glucose
TC: Total cholesterol
HDL-c: High-density lipoprotein cholesterol (HDL-c)
AUROC: The area under the receiver operating characteristic curve
NAFLD: Non-alcoholic fatty liver disease
BMI: Body mass index
AIP: Atherogenic index of plasma
