# Supplementary material for: The value of the atherogenic index of plasma in non-obese people with non-alcoholic fatty liver disease: a secondary analysis based on a cross-sectional study
Source: Lipids Health Dis. 2020 Jun 23;19:148. doi: 10.1186/s12944-020-01319-2 (PMC7313140; doi:10.1186/s12944-020-01319-2)
Supplement: Supplementary file 1 — Additional file 1: Table S1. Baseline Characteristics of the Japanese Study Participants. Table S2. Effect of AIP on the incidence of NAFLD in Japanese. Table S3. AUROC analysis for different continuous predictors in Japanese groups. Figure S1. The relationship between AIP and NAFLD. Figure S2. AUROC curve of all risk factors in the Japanese group. [file 12944_2020_1319_MOESM1_ESM.docx]

| Table S1 Baseline Characteristics of the Japanese Study Participants | | | |
| --- | --- | --- | --- |
|  | Non-NAFLD | NAFLD | *P*-value |
| No. of participants | 11598 | 1334 |  |
| age | 43.38 ± 9.03 | 45.86 ± 8.33 | < 0.001 |
| GGT (U/L) | 14.00 (11.00,19.00) | 22.00 (16.00, 31.00) | < 0.001 |
| ALT (U/L) | 15.00 (12.00, 20.00) | 24.00 (18.00, 33.00) | < 0.001 |
| AST (U/L) | 17.39 ± 8.16 | 20.63 ± 7.63 | < 0.001 |
| HDL-c (mmol/L) | 1.54 ± 0.40 | 1.24 ± 0.32 | < 0.001 |
| BMI (kg/m^2)^ | 20.89 ± 2.09 | 23.09 ± 1.40 | < 0.001 |
| AIP | -0.38 (-0.58, -0.16) | -0.02 (-0.21, 0.18) | < 0.001 |
| TG (mmol/L) | 0.64 (0.45,0.93) | 1.15 (0.84, 1.66) | < 0.001 |
| FPG (mmol/L) | 5.09 ± 0.40 | 5.37 ± 0.37 | < 0.001 |
| Sex |  |  | < 0.001 |
| Female | 6169 (53.19%) | 237 (17.77%) |  |
| Male | 5429 (46.81%) | 1097 (82.23%) |  |

| Table S2 Effect of AIP on the incidence of NAFLD in Japanese | | | | | | |
| --- | --- | --- | --- | --- | --- | --- |
| Variable | Unadjusted | | Adjusted Model I | | Adjusted Model II | |
|  | OR (95%CI) | *P*-value | OR (95%CI) | *P*-value | OR (95%CI) | *P*-value |
| AIP (Per SD) | 31.2 (25.6, 38.2) | < 0.001 | 18.8 (15.2, 23.3) | < 0.001 | 10.8 (6.8, 17.3) | < 0.001 |
| AIP (quartile) |  |  |  |  |  |  |
| Q1 | Reference |  | Reference |  | Reference |  |
| Q2 | 3.3 (2.3, 4.7) | < 0.001 | 2.7 (1.9, 3.9) | < 0.001 | 1.8 (1.2, 2.6) | < 0.001 |
| Q3 | 8.8 (6.3, 12.3) | < 0.001 | 6.0 (4.3, 8.5) | < 0.001 | 3.1 (2.2, 4.5) | < 0.001 |
| Q4 | 29.5 (21.3, 40.8) | < 0.001 | 17.2 (12.3, 24.1) | < 0.001 | 4.8 (3.3, 7.0) | < 0.001 |
| *P* for trend | < 0.001 | | < 0.001 | | < 0.001 | |

Model I adjusted for age, sex

Model II adjusted for sex, age, GGT, ALT, AST, BMI, TG, FPG.

CI: confidence interval.

| Table S3 AUROC analysis for different continuous predictors in Japanese groups | | | | | |
| --- | --- | --- | --- | --- | --- |
| Predictors | AUROC | 95%CI | Cut-off pot | Specificity | Sensitivity |
| GGT (IU/L) | 0.749 | 0.736-0.762 | 16.500 | 0.670 | 0.729 |
| ALT (IU/L) | 0.798 | 0.786-0.811 | 20.500 | 0.783 | 0.667 |
| AST (IU/L) | 0.650 | 0.634-0.666 | 18.500 | 0.653 | 0.568 |
| HDL-c (mmol/L) | 0.736 | 0.722-0.749 | 1.400 | 0.604 | 0.761 |
| BMI (kg/m^2^) | 0.804 | 0.794-0.815 | 21.677 | 0.626 | 0.849 |
| TG (mmol/L) | 0.783 | 0.771-0.796 | 0.841 | 0.694 | 0.744 |
| FPG (mmol/L) | 0.700 | 0.686-0.714 | 5.135 | 0.562 | 0.735 |
| AIP | 0.798 | 0.787-0.810 | -0.220 | 0.695 | 0.768 |


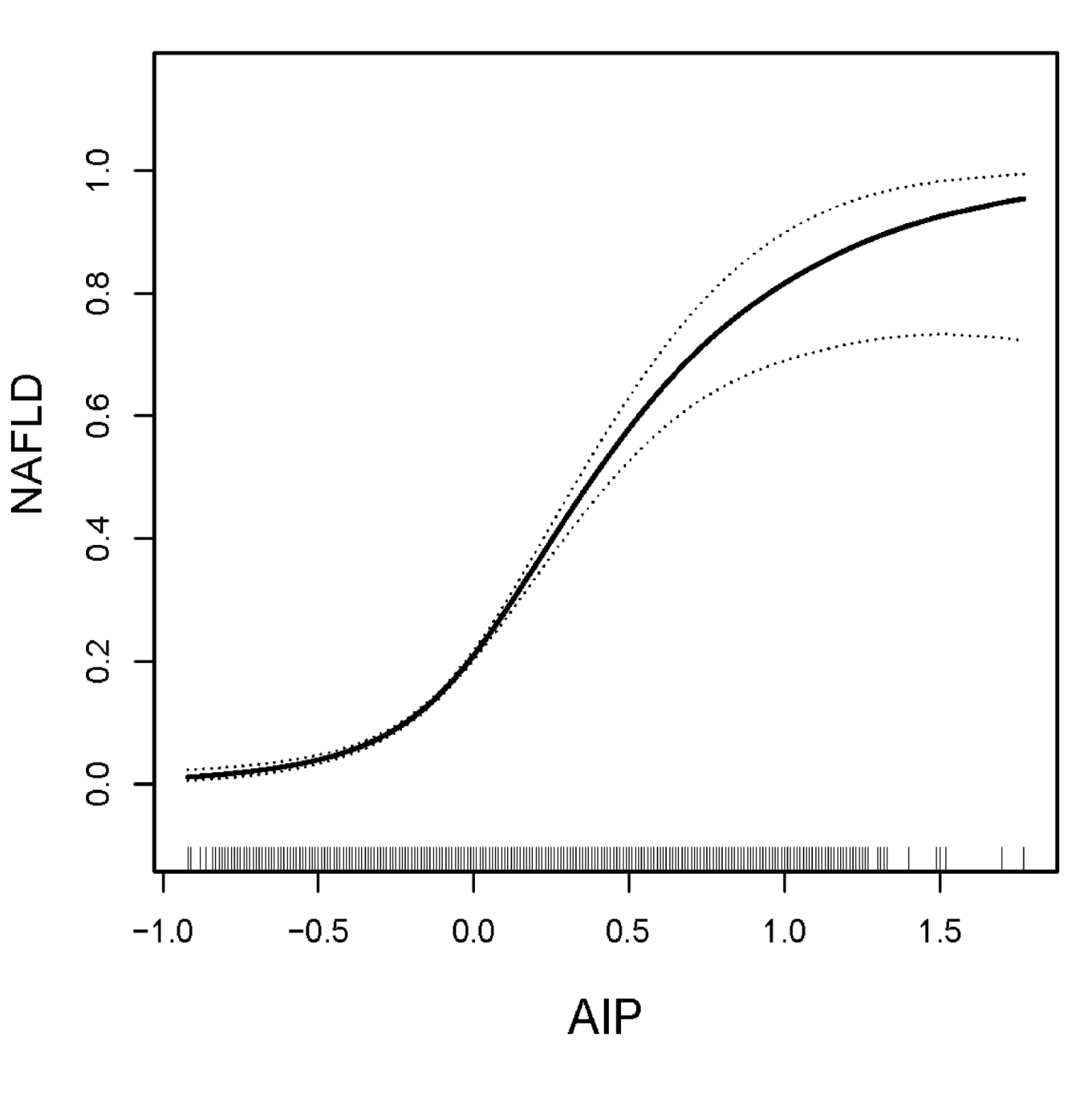


Fig.S1 The relationship between AIP and NAFLD


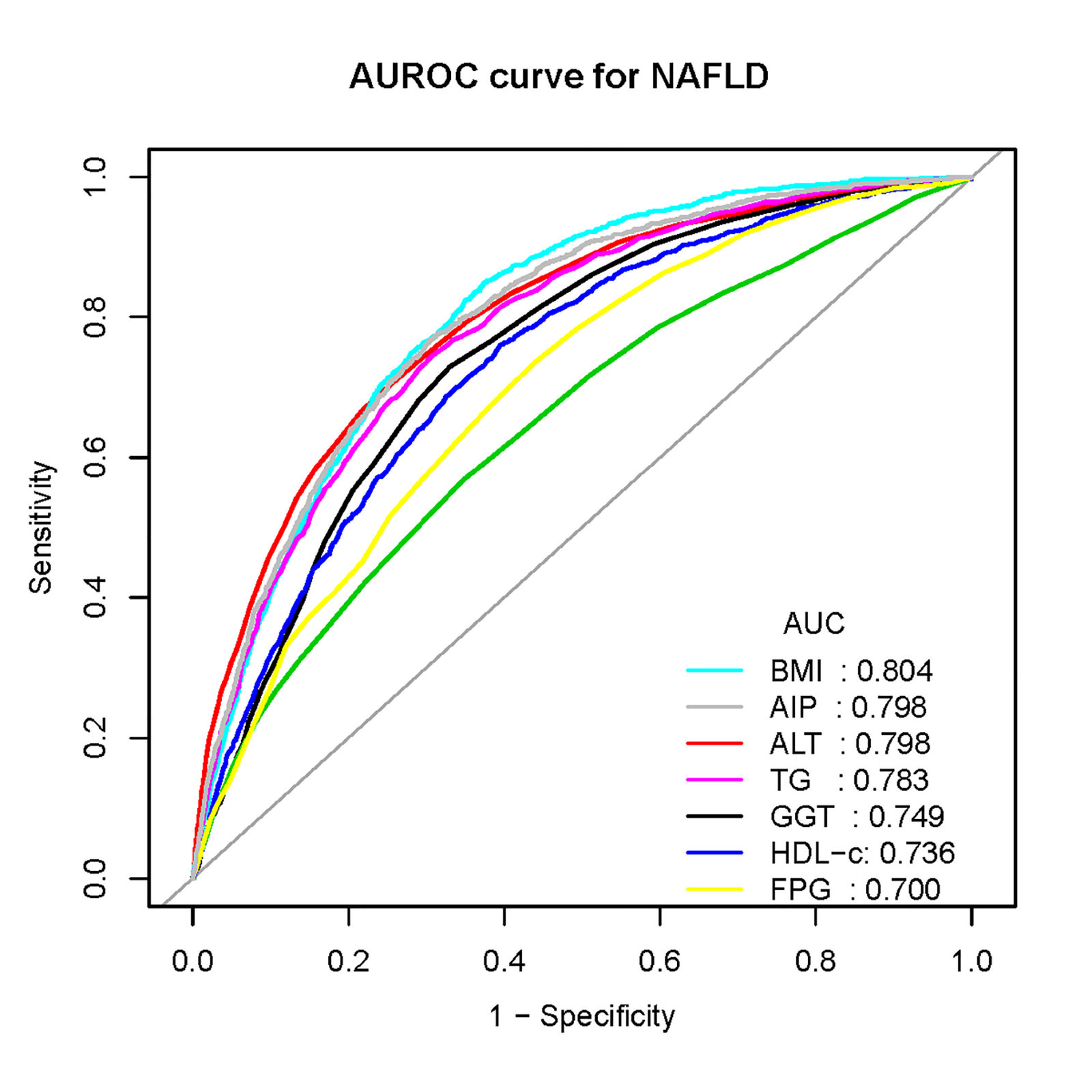


Fig.S2 AUROC curve of all risk factors in the Japanese group
